# Digital Spindle: A New Way to Explore Mitotic Functions by Whole Cell Data Collection and a Computational Approach

**DOI:** 10.3390/cells9051255

**Published:** 2020-05-19

**Authors:** Norio Yamashita, Masahiko Morita, Hideo Yokota, Yuko Mimori-Kiyosue

**Affiliations:** 1 Image Processing Research Team, RIKEN Center for Advanced Photonics, 2-1, Hirosawa, Wako, Saitama 351-0198, Japan; norio_yamashita@riken.jp (N.Y.); mmorita@riken.jp (M.M.); hyokota@riken.jp (H.Y.); 2 Laboratory for Molecular and Cellular Dynamics, RIKEN Center for Biosystems Dynamics Research (BDR), 2-2-3 Minatojima-minamimachi, Chuo-ku, Kobe 650-0047, Japan

**Keywords:** 3D live imaging, lattice light-sheet microscopy, mitosis, mitotic spindle, information science

## Abstract

From cells to organisms, every living system is three-dimensional (3D), but the performance of fluorescence microscopy has been largely limited when attempting to obtain an overview of systems’ dynamic processes in three dimensions. Recently, advanced light-sheet illumination technologies, allowing drastic improvement in spatial discrimination, volumetric imaging times, and phototoxicity/photobleaching, have been making live imaging to collect precise and reliable 3D information increasingly feasible. In particular, lattice light-sheet microscopy (LLSM), using an ultrathin light-sheet, enables whole-cell 3D live imaging of cellular processes, including mitosis, at unprecedented spatiotemporal resolution for extended periods of time. This technology produces immense and complex data, including a significant amount of information, raising new challenges for big image data analysis and new possibilities for data utilization. Once the data are digitally archived in a computer, the data can be reused for various purposes by anyone at any time. Such an information science approach has the potential to revolutionize the use of bioimage data, and provides an alternative method for cell biology research in a data-driven manner. In this article, we introduce examples of analyzing digital mitotic spindles and discuss future perspectives in cell biology.

## 1. Introduction

Capturing the dynamic processes of intracellular events at the whole cell level is needed to understand how cells coordinate various systems, including cytoskeletons, cell adhesions, and membrane trafficking. However, insufficient performance in the resolution, speed, and depth of conventional imaging techniques has crucially limited what we can observe [1,2]. For example, in the mitotic apparatus, which is comprised of hundreds of microtubule filaments and plays an essential role in cell division [3,4,5], direct observation of individual filaments has never been achieved [6,7]. Because numerous microtubule-regulating factors work in cooperation to ensure the correct segregation of chromosomes into each daughter cell by finely tuning the dynamics of individual filaments [8,9,10], the parameters of microtubule dynamics are crucial to monitor mitotic functions. 

**Figure 1:**
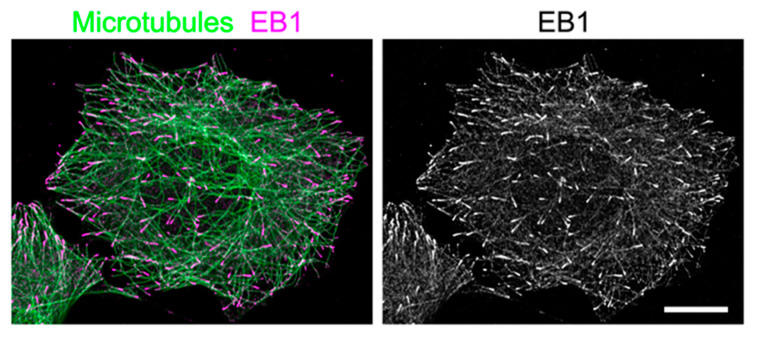
Distribution of end-binding 1 protein (EB1) in a flat, interphase HeLa cell. HeLa cells were fixed and immunostained for microtubules (green) and EB1 (magenta), and visualized by conventional confocal microscopy (LSM780, Carl Zeiss) to show EB1 localization at microtubule ends. In the right image, only EB1 signals are shown in grayscale. Scale bar: 5 μm.

Recently, we detected microtubule growth dynamics in three dimensions at the whole-cell level, using recently developed lattice light-sheet microscopy (LLSM), which enables three-dimensional (3D) scanning at subsecond intervals with high spatial resolution [11,12], in conjunction with microtubule growth marker protein end-binding 1 (EB1), a microtubule plus-end-tracking protein, which was fused to green fluorescent protein (EB1–GFP) [13]. EB1–GFP binds selectively to the growing ends of microtubules as a comet shape pattern, with a short axis diameter of approximately 25 nm and an anteroposteriorly elongated tail of up to 500 nm (Figure 1), which has shown its utility as an analytical tool to study microtubule growth in cells and animals [14,15,16,17]. Time-lapse imaging and tracking of EB1–GFP comets allow the generation of microtubule growth trajectories, which allows for the analysis of the microtubule growth rate, where the growth occurred, and its direction [14,18]. However, until recently, image acquisition and analysis had been predominantly limited to a two-dimensional (2D) plane, because the spatiotemporal resolution of conventional fluorescence microscopes is insufficient to follow the 3D motion of EB1–GFP comets. However, by obtaining high-resolution images of whole cells with LLSM, the current challenge is the need for technology development to analyze the large amounts of data.

Modern, live-cell 3D microscopy, such as LLSM, is now providing vast amounts of data that can no longer be handled by conventional approaches, which depend on human eyes and manual editing. This is a challenge that cell biology has never experienced before, and efforts to address it have just begun. Based on our own experiences in the analysis of mitosis data obtained by LLSM, we discuss the issues that need to be resolved and the future of big image data analysis that will reform research approaches in cell biology. 

## 2. High Spatiotemporal Resolution, Whole-Cell, 3D Live Imaging by LLSM

### 2.1. Principles of LLSM

**Figure 2:**
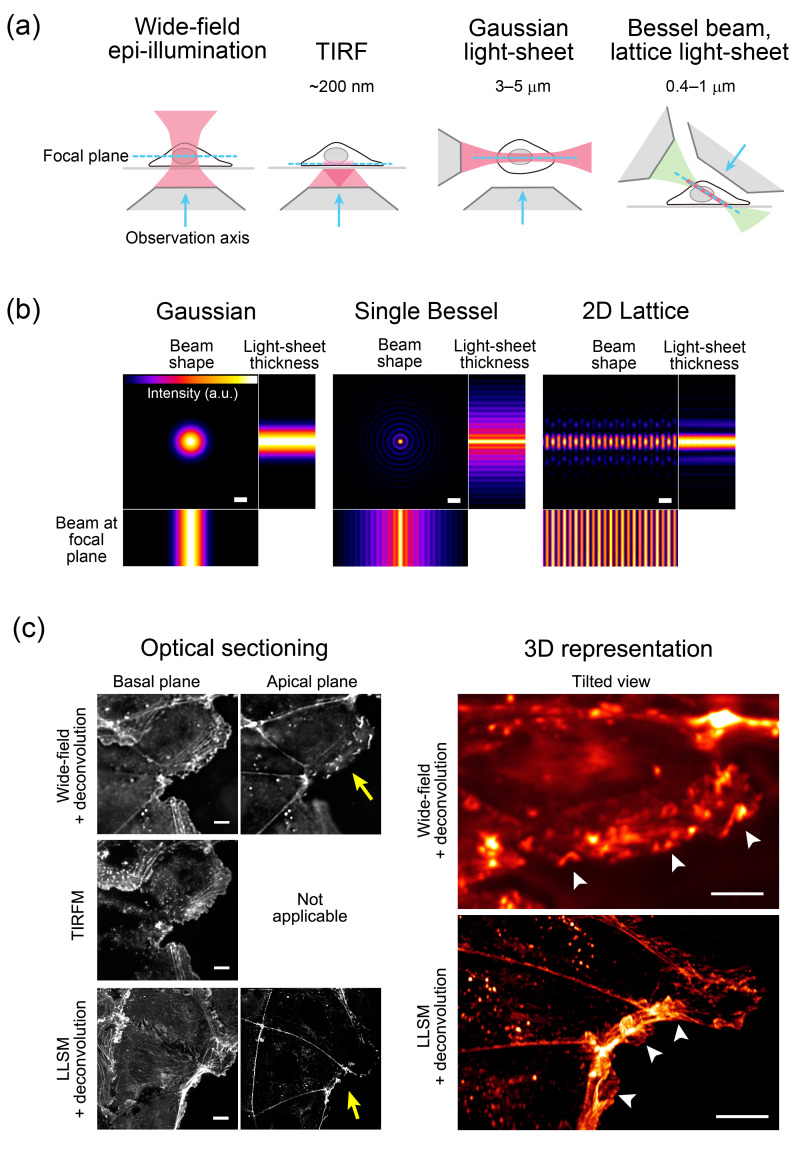
Comparisons of the optical sectioning power and beam shapes used in different illumination techniques. (**a**) Optical sectioning power of wide-field epi-illumination, total internal reflection fluorescence (TIRF), Gaussian light-sheet, and Bessel beam/lattice light-sheet techniques. Depth (TIRF) and thickness (light-sheet) of irradiation areas are shown. (**b**) Beam shapes of three different illumination techniques. A Gaussian beam is used in traditional laser-scanning light-sheet microscopy [20]. To create a virtual light-sheet, the beam is swept rapidly, providing time-averaged uniform illumination. A multi-Bessel beam increases the sample scanning speed. Adapted from [11]. Scale bars: 1 µm. (**c**) Comparison of optical sectioned images of human colon carcinoma (Caco2) cells expressing Lifeact–red fluorescent protein (RFP) visualizing actin filaments [21], collected by wide-field, TIRF microscopy (TIRFM), and lattice light-sheet microscopy (LLSM) (left column). In wide-field and TIRFM, the same field of view was imaged using a TIRFM system (Nikon). Three-dimensional stacks of wide-field and LLSM images were prepared for deconvolution. Resliced images of LLSM were generated using the 3D Volume Viewer function of ImageJ. Optical sections at the basal plane showing cell–substratum attachment and the apical plane visualizing cell–cell adhesions are shown. Note that only the basal image is available in TIRFM. The TIRFM image shows high contrast signals at the basal plane, whereas the LLSM images show high contrast, high resolution signals at both basal and apical planes. In the right column, wide-field and LLSM images are represented as three-dimensional (3D) and viewed from a tilted angle to observe membrane ruffling (arrow heads) using Imaris software (Bitplane). The approximate direction of observation is indicated by the yellow arrow on the left. Scale bars: 10 µm. See also Video S1 showing the tilted view of the images.

LLSM, which enables ultrafast 3D scanning with high spatial resolution [11], is an advanced type of light-sheet microscopy [19]. In contrast to a conventional light-sheet, created by several micron-thick Gaussian beams, the lattice light-sheet is generated by a massive parallel array of non-diffracting light beams (Bessel beams) that mutually interfere to create an ultrathin light-sheet of 0.4–1 μm thick extending over cellular dimensions [11] (Figure 2a,b). Scanning with the ultrathin light-sheet reduces fluorescence photobleaching and phototoxicity, obtaining images of whole cells for hundreds of volumes at subsecond intervals at the resolution of ~230 nm laterally and ~370 nm axially [11].

A Bessel beam is a special class of non-diffracting beam that allows a narrow beam width to propagate without spreading over a long distance, because of the self-interference effect of the beam [22,23,24] (Figure 2b). A Bessel beam can be obtained as diffracted light at the center of inclined beams generated using a conically shaped special lens, known as an axicon. Even if there is an obstacle on the optical axis, the Bessel beam can be generated behind it. Thus, a Bessel beam is also called a self-reconstructing beam [25]. In LLSM, multiple Bessel beams are generated using a programmable spatial light modulator [11]. The spacing of the beams is tightly controlled, in order to allow them to interact with each other constructively in the central plane to form an ultrathin, 2D light-sheet [2,11] (Figure 2a,b). 

The most prominent feature of light-sheet illumination is the axial confinement of illumination rather than needlessly irradiating the entire volume, which dramatically reduces photobleaching and phototoxicity, allowing long-term imaging [1,19,20,24,26,27,28,29,30]. Simultaneously, the ultrathin (0.4–1 μm) light-sheet of LLSM yields a superior signal-to-background ratio comparable to total internal reflection fluorescence microscopy (TIRFM), even in thick, fluorescently dense specimens [2] (Figure 2c, Video S1). Another important feature of the light-sheet method is high-speed imaging by detecting the entire field of view of the in-focus plane [1,19,24,26]. LLSM achieves a plane-wise imaging rate as high as >200 frames per second, and whole-cell 3D scanning often at subsecond intervals [11].

### 2.2. Image Collection by LLSM

LLSM offers two imaging modes: a super resolution-structured illumination microscopy (SIM) mode and a high-speed dithered mode [11]. In the SIM mode, collecting multiple images at each *z*-plane, >200 3D volumes can be acquired at 4 s intervals at the 150 × 280 nm *xz* resolution. In the dithered mode, the 2D lattice pattern is oscillated back and forth using a galvanometer, providing time-averaged uniform illumination; only one 2D image at each *z*-plane is acquired, at up to 100–200 frames per second for thousands of time points, at the resolution of 230 nm in *x* and ∼370 nm in *z*. This is ∼1.3–1.5 times poorer in each direction than the SIM mode, but dithered lattices allow for ∼7.5 times faster imaging at a comparable signal-to-noise ratio. Furthermore, the dithered mode collecting single plane images at each *z*-plane minimizes photobleaching and phototoxicity, in contrast to the SIM mode collecting multiple z-planes to create a single slice image. Overall, the dithered lattice light-sheet is advantageous for most biological systems, unless the additional resolution of the SIM mode is required.

### 2.3. LLSM with Adaptive Optics (AO-LLSM)

The later model of LLSM with adaptive optics (AO), which correct for sample-induced aberrations in multicellular specimens [31,32,33,34], has been used to visualize mitotic processes during zebrafish embryogenesis [35]. AO-LLSM uses two-channel AO to correct aberrations at both excitation and detection paths by scanning the reference “guide star” created through two-photon excited fluorescence over the region to be imaged. In this system, the spatiotemporal resolution and non-invasiveness are comparable to the original LLSM approach, but tiled acquisition collects a larger volume, such as a >200 × >200 × >100 µm block covering a wide range of developing tissues. 

One of the limitations of the original model is that the movable range of the sample stage is relatively narrow, and samples must be smaller than the 5 mm-diameter glass coverslip. With AO expanding the observable area, the imaging chamber has been modified to allow the loading of larger specimens. This modification was effective even without AO when observing the entire *Drosophila* brain, and across the width of the mouse brain cortex subjected to expansion microscopy [36], in which proteins are anchored to a swellable gel to a create expanded, optically clear phantom of a fluorescent specimen that retains its original relative distribution of fluorescent tags [37]. 

## 3. Image Data Processing and Analysis

### 3.1. Whole-Cell 3D Movie of Mitosis and Generation of Digital Spindles

**Figure 3:**
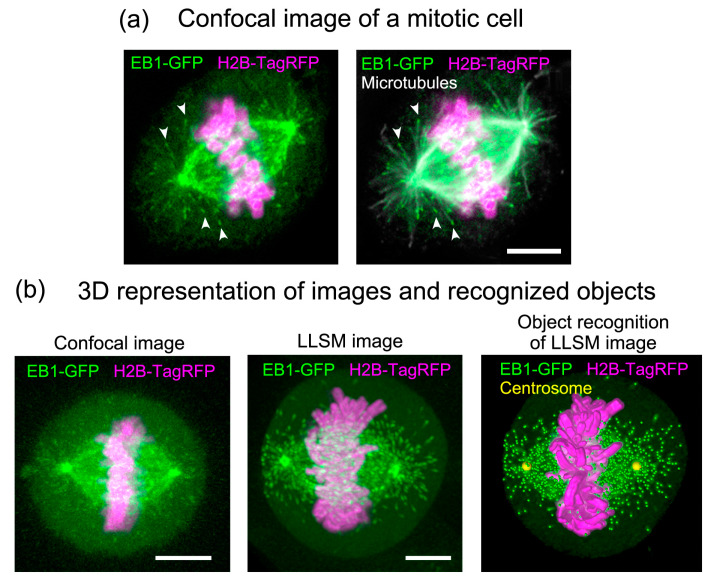
Comparison of 3D stack images collected by conventional confocal microscopy and LLSM. (**a**) Distribution of EB1– green fluorescent protein (GFP) in a mitotic HeLa cell at metaphase. HeLa cells (clone A1) stably expressing EB1–GFP (green) and histon H2B–TagRFP (magenta) [11] were fixed and immunostained for microtubules (white) to identify the positions of microtubule ends. Z-stack images were collected under a conventional laser scanning confocal microscope (LSM780, Carl Zeiss). Note that during mitosis, the cells become round and thicker than cells in interphase. Therefore, the signal is blurred and does not appear as sharp as during interphase, as shown in Figure 1. Scale bar: 5 μm. Adapted from [12]. (**b**) Images collected by confocal microscopy (left) and LLSM (middle) with comparable *xyz* pixel pitches were displayed in a 3D space using Imaris software (Bitplane). Confocal imaging was performed using fixed cells, because it requires a long scan time, whereas LLSM imaging was performed using living cells. On the right, the positions of the EB1-GFP comets (green dots), centrosomes (yellow dots), and surface rendering of chromosomes (magenta) are superimposed on the original image (middle). Scale bars: 5 μm.

The remarkable improvement in *z*-resolution of LLSM provides, for the first time, a complete overview of whole mitotic cells as time-lapse videos with submicron 3D resolution, which can be observed from any viewing angle with similar resolving power by rotating the image [11,12]. This 3D representation of the data clearly demonstrates its advantage over the conventional laser confocal method (Figure 3b).

Using EB1–GFP as a microtubule growth marker [13,14], we detected microtubule growth trajectories throughout the mitotic cell volume, including the inside of spindles [12] (Video S2). Prior to the tracking of EB1–GFP comets, drift correction was applied for the spindle position, because the spindle apparatus serves as a useful frame of reference during cell division; however, it often rotates and changes orientation during division. In the time-lapse sequence collected at 0.755 s intervals over a 56.625 s duration (75 frames), >10,000 EB1–GFP comets and >2000 trajectories were detected in each mitotic cell (Video S2). 

**Figure 4:**
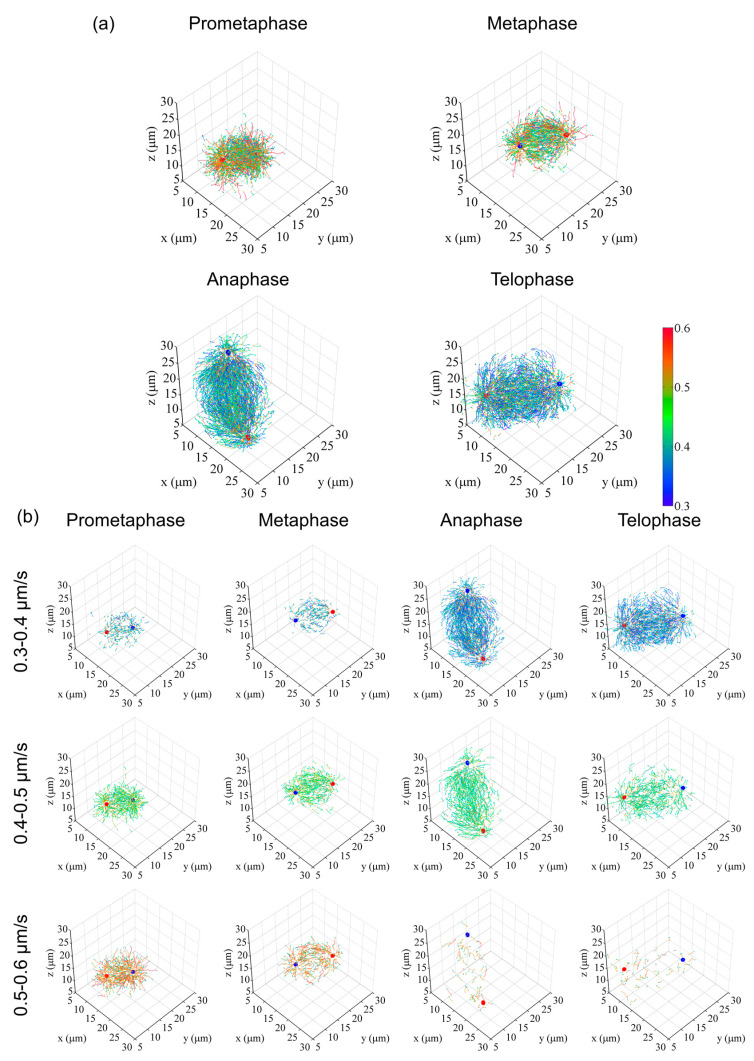
Examples of digital spindle analysis. (**a**) Digitized and 3D-represented microtubule growth trajectories in various mitotic phases, represented using custom tools created in Matlab. For original images and EB1–GFP tracking to generate trajectories, see also Video S2. Three cell data (prometa, meta, ana) and two cell data (telo) were merged to generate average models. To improve visibility, trajectories were randomly extracted at approximately 13% per cell (prometa, meta, ana) or 20% per cell (telo). Red and blue dots indicate centrosome position. Orange and cyan dots indicate the start and end position of trajectories, respectively. The colored bar indicates the range of the mean travel speed of EB1–GFP comet trajectories (0.3–0.6 µm/s). (**b**) Merged trajectories were divided into 10 classes spanning the entire speed data range (0–1 µm/s, 0.1 µm/s steps). Data included in the 0.3–0.6 µm/s range are shown. Markers are similar to those in (**a**). Data are reused from [12].

**Figure 5:**
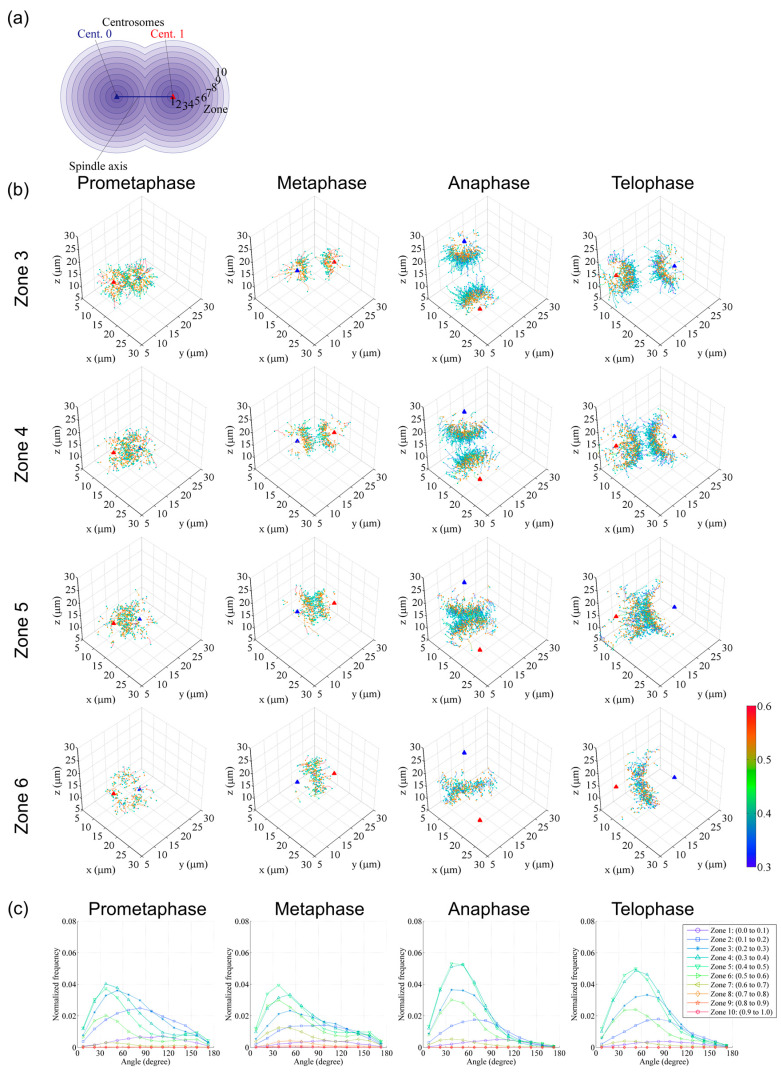
Examples of digital spindle analysis (continued). (**a**) To classify trajectories according to the start position, a spherical classification bin (zone) was set. Spheres centered at centrosomes (red and blue triangles) and with radii equal to the intercentrosomal distance were generated, and then divided into 10 spherical zones of equal radial length. (**b**) Trajectories starting at each zone were extracted and plotted in 3D coordinate systems. The trajectories are shown with colors corresponding to the average speed of each trajectory, with orange dots at the start of the trajectory and cyan dots at the end. The colored bar indicates the mean speed (µm/s). (**c**) The travel angle of each comet against the spindle axis was analyzed and plotted for each zone. Data are reused from [12]. Plots in (**b**,**c**) were generated using custom tools created in Matlab.

Once the data are digitized as the coordinate information of objects of interest, the objects can be processed computationally for selection, classification, or the grouping of objects and geometric presentation for the interpretation of volumetric data. For example, in Figure 4 and Figure 5, trajectories were classified by growth speed and the position of the first track point, respectively, and displayed in the *x*-*y*-*z* coordinate system. 

In Figure 4b, trajectories are grouped with mean travel speeds into 0.1 µm/s interval bins and displayed separately for each range in 3D coordinate systems. This representation clearly visualizes the spatial shift in the distribution of trajectories with different growth rates as cell division progresses. In Figure 5b, trajectories were arranged into 10 spherical zones, as shown in Figure 5a, and trajectories of various data bins were then presented separately. These data were also used to analyze the trajectory angle between the microtubule growth direction and spindle axis (Figure 5c). These examples demonstrate that such visualization of information is useful and essential to interpret large and complex image datasets.

### 3.2. Cell Division in Developing Embryos

AO-LLSM has been applied to study various 3D subcellular processes throughout the cell cycle, across populations of cells in vivo and in organoids [35]. The data unveiled changes in the organization and position of organelles as cell division progressed, the volume of cells and contained organelles, and the synchrony of cell division timing. Observation of different organs in zebrafish embryos revealed the phenotypic diversity across different cell types and developmental stages, and similarities and differences in mitosis progression observed in cultured cells. For example, the formation of plasma membrane blebs before cytokinesis observed in culture [38,39] was commonly observed in various zebrafish organs.

### 3.3. Digital Data Archiving and Its Utilization

As shown above, the high spatiotemporal resolution images of whole cells collected by LLSM are a rich source of information, and can be used any time for reanalysis of data for various purposes. This is possible because LLSM collects precise information from all over the cell without bias arising from limited and incomplete data. Previously because of the performance limitations of microscopes, the experimenters had to perform live imaging of fast-moving targets by focusing on a certain optical plane that they found desirable. This restriction causes experimenter bias, and conventional microscopy images with limited dimensions cannot contain enough information to contribute to objective information analysis. In contrast, the information obtained exhaustively throughout the cell by LLSM and scanned without bias, such as selection of the focus plane by human judgment, which is necessary to establish a generic model, warrants analysis by advanced information science techniques, in order to extract information that cannot be recognized by visual inspection. 

To make such approaches efficient and feasible, dataset sharing would be effective, so that data can be analyzed by other researchers for different purposes and from different perspectives [35]. These approaches have been adopted in developmental biology, using light-sheet microscopy optimized for embryonic development by releasing digital images/data showing the developmental processes of early embryos, such as zebrafish and *Drosophila*, which are stored in a database [20,40,41]. Additionally, in the cell biology field, not only for mitosis research, but also various cell function studies, sharing datasets containing accurate and sufficient information will change the research style of cell biology. In this case, it is even more preferable that the data are provided on a large-scale cloud computing infrastructure together with calculation functions, so that a large amount of calculative processing can be performed remotely without high calculation capability and highly specialized knowledge at the biologist’s location. 

### 3.4. Big Image Data Problem

To this end, though, because image analysis by extensive manual editing of numerous image series containing high spatiotemporal information collected by LLSM is very time consuming, and the pipeline data processing requires extensive calculations, it is difficult to process a larger number of datasets routinely. LLSM technology generates large amounts of image data, easily reaching 200 GB to 1 TB for each time-lapse, and thousands of numerical datasets contain far more information than can be practically analyzed by conventional approaches. Therefore, automated computational strategies are essential to extract biologically significant information in an efficient and reproducible manner. 

Recently, Cai et al. reported the generation of a dynamic protein atlas of human cell division [42]. They automatically imaged mitotic cells in culture using a conventional confocal microscope, followed by calibration of the signal by fluorescence correlation spectroscopy to convert 3D protein fluorescence movies to time-resolved distribution maps of protein concentrations. Using the data, they generated a canonical model of the morphological changes during mitotic progression. Because LLSM generates a significant amount of data, including more high-resolution information, applying such an approach to time-series volume data may provide new opportunities for big image data mining. 

In information science, one of the most successful advances in big data analysis in recent years has been deep learning technologies. Previously, an unsupervised learning method based on temporally constrained combinatorial clustering for the automatic prediction of cell morphology classes in 2D time-lapse images was reported [43]. Very recently, Driscoll et al. developed a generic morphological motif detector for 3D images, which automatically finds lamellipodia, filopodia, blebs, and other motifs using u-shape3D, a computer graphics and machine-learning pipeline [44]. Extending this technology to analyze a variety of other cellular structures would be a promising direction.

In the case of AO-LLSM, when collecting high-resolution data over a large area, image visualization is the primary challenge, because it is impossible to observe what is inside due to the overlapping of numerous images containing fine and dense patterns. To solve this problem, a method has been developed to computationally separate the distance between individual cells for observation inside [35]. One of the important features of multicellular systems is diversity. So far, no technology has been developed to automatically find and classify different cell types and their internal structures in multicellular tissues, and automatically analyze dynamic changes in their morphology and activity. Now that the high-resolution observation of multicellular systems has been achieved, automatic detection and analysis of differences between cells, which cannot be detected using conventional technologies at both the data collection and analysis stages, will be the next major challenge. 

## 4. Conclusions and Future Perspectives

Recent rapid developments in live-cell 3D microscopy have begun to enable the imaging of cell morphology and signaling with unprecedented detail. These imaging techniques facilitate innovative life science, including comprehensive quantitative analysis of cellular functions in development and diseases, as well as high-throughput, high content screening for drug discovery. In fact, light-sheet microscopy has contributed to not only the discovery of principles of early development, as described above [20,28,29,41], but also to biomaterial development, tissue engineering, and regenerative medicine. For example, the movement and morphology of mesenchymal stem cells and tumor cells over the surface of a spherical microcarrier [45,46,47] and the differentiation and connection with host neurons of transplanted human cells in whole mouse brain [48,49] were visualized to advance medical applied research. 

A common issue that has become apparent with the development of microscopy technology is the big data problem. No tools or standardized methodologies for big image data analysis exist yet. Although traditionally, fewer software tools have existed in biological laboratories than optical technologies [50,51], cell biology now requires closer collaboration with information science than ever before. Once a functional environment for big image data analysis has been established, digital cellular models derived from 3D microscopies, including LLSM, that provide accurate and sufficient information will be powerful tools to reverse engineer cellular processes for the discovery of novel mechanisms, targeted research of molecular mechanisms using cell profiling techniques, and drug screening with a large amount of highly accurate information as examples. 

This will be facilitated by integrating microscopic technologies with newly emerging technologies in different fields. For example, to prepare specimens mimicking normal development and disease states, cellular/tissue engineering technologies, such as 3D organoid culture [52,53], artificial tissues using moldable transparent hydrogel material [54,55], and organ-on-a-chip devices [56,57] to reproduce cellular behavior in vivo have great potential. In addition, multiplexed labeling technology is useful to detect a large number of molecules in each cell [58,59,60,61,62,63]. By repeating the elution and reloading of fluorescently labeled antibodies [58] or proteins [62] in fixed and permeabilized cells, the detection of more than 40 different proteins has been achieved. Automation of imaging and analysis processes was the key to successful practical application of these techniques [58,59,60]. Similar procedures can be applied to 3D specimens in combination with high speed 3D imaging. The improved accuracy and reliability of data, faster scanning, and automation by the optimal combination of emerging technologies in various fields (not limited to the imaging field including optics and probes) will establish the foundation for next-generation cell biology research.

## Supplementary Materials

The following are available online at https://www.mdpi.com/2073-4409/9/5/1255/s1, Video S1: Tilted view of 3D images of Caco2 cells expressing Lifeact-RFP visualizing actin filaments, by wide-field and LLSM; Video S2: Time-lapse images of EB1–GFP at different phases during mitosis and EB1–GFP tracking to generate microtubule growth trajectories.
